# Synthesis of Modified Starch/Polyvinyl Alcohol Composite for Treating Textile Wastewater

**DOI:** 10.3390/polym12020289

**Published:** 2020-02-01

**Authors:** Kai Xia, Xin Liu, Weiwei Wang, Xizi Yang, Xiaodong Zhang

**Affiliations:** College of Chemistry and Chemical Engineering, Qingdao University, Qingdao 266071, China; xiakaiqd@163.com (K.X.); liuxinqdu@126.com (X.L.); weiweiqdu@163.com (W.W.); xiziy91@163.com (X.Y.)

**Keywords:** composite microspheres, modified polyvinyl alcohol, modified starch, textile wastewater, regenerated activated carbon

## Abstract

In this work, we demonstrated a strategy to design a modified starch/polyvinyl alcohol composite (CCSP), which was employed as a highly efficient and economical fixed-bed adsorbent for treating textile wastewater. Characterization revealed that most of the CCSP was shaped with the morphology of sphericity, and had some water swelling properties. The crystallinity of the CCSP was lower than that of native starch and polyvinyl alcohol, and its average particle size gradually increased with the dosage increase of cationic starch in the preparation. Adsorption experiments showed that the adsorption capacities of CCSP were more than 605 and 539 mg/g for Reactive Black 5 and Reactive Orange 131, respectively, which were over 10 times larger than that of commercial activated carbon (AC). The mixture adsorbent composed of CCSP and AC could remove starch, polyvinyl alcohol, and dyes from textile wastewater completely and simultaneously combined with the fixed-bed technique, and its adsorption capacity was conducted as a function of the bed height and flow rate. Most importantly, the disabled mixture adsorbent could be converted into regenerated AC through a chemical activation process, thereby avoiding the production of solid waste. This study will provide a new efficient green sustainable method for treating textile wastewater.

## 1. Introduction

The environmental problems caused by textile printing and dyeing wastewater have become the focus of attention. Typical textile printing and dyeing wastewater usually contains dyes, polyvinyl alcohol (PVA), starch, and so on [1,2]. Some dyes may be further degraded into toxic and carcinogenic substances, which will affect human health [3]. The efficient and complete removal of dyes from effluents is necessary. Starch and PVA in textile printing and dyeing wastewater have a great influence on the reoxygenation behavior of bodies of water. As a result of the high chemical oxygen demand (COD) values and lower biodegradability, PVA is difficult to remove from textile wastewater by simple treatment facilities [4,5]. A lot of technologies, including flocculation, membrane separation, chemical oxidation, and degradation, have been studied for treating dye wastewater [6,7,8,9] or for treating PVA-containing wastewater [5,10,11,12,13], but so far, there have been no reports on the simultaneous removal of dyes, PVA, and starch from textile printing and dyeing wastewater. These methods also have many difficulties for industrial application, due to the high cost of investment and operation, complicated processes, and inability to remove pollutants completely [5,12,14]. By contrast, adsorption is one of the most efficient and reliable textile wastewater treatment techniques [15,16,17], and fixed-bed adsorption is often desired from an industrial point of view, because of its advantages of its 100% pollutant removal ability from wastewater, lower floor space, and lower cost of equipment investment and operation [18,19,20,21,22]. Many studies have been conducted on the dye adsorption properties of various adsorbents used in fixed beds, such as agricultural waste, chitosan-based materials, zeolites, and industrial by-products, etc. [18,23,24,25]; however, these adsorbents usually have defects with a low adsorption capacity. Up until now, activated carbon (AC), although its removal efficiencies for organic dyes are still relatively low [26,27], is still the most commonly used adsorbent for industrial wastewater treatment [28,29]. Therefore, in order to remove dye, polyvinyl alcohol, and starch from textile wastewater simultaneously, it is necessary to develop an efficient and low-cost fixed-bed adsorbent alternative.

Starch and its derivatives are of increasing interest because of their higher biodegradability and renewability [30,31]. Cationic starch derivatives with quaternary ammonium groups are commonly prepared through the reaction of starch with 3-chloro-2-hydroxypropyltrimethylammonium chloride (CHPTAC) [32,33]. As a result of the strong quaternary ammonium groups, cationic starch derivatives can adsorb anionic compounds, and have been widely utilized for removing various ions and organic pollutants from wastewater [34,35,36]. PVA possesses hydrophilic, biocompatible, and nontoxic characters. PVA derivatives are important functional polymers, and are widely used in paper, medicine, and water treatment [37,38,39]. Nevertheless, so far, there have been no reports on the synthesis of modified starch/PVA composite microspheres and their applications in textile wastewater treatment.

In this paper, the cationic cross-linked starch/PVA composite microspheres (CCSP) were synthesized for the purpose of removing the starch, PVA, and dyes from the textile wastewater concurrently, and their properties were determined. Combined with fixed-bed adsorption techniques, the adsorption capacities of CCSP for treating textile wastewater were investigated. Most importantly, the CCSP used exhibited great sustainable reutilization by converting into regenerated AC, thereby avoiding secondary solid-waste pollution. It is expected that the strategies shown in this work will provide new inspiration and ideas for the development of environmentally-friendly textile wastewater treatment techniques.

## 2. Materials and Methods 

### 2.1. Materials

**Figure 1:**
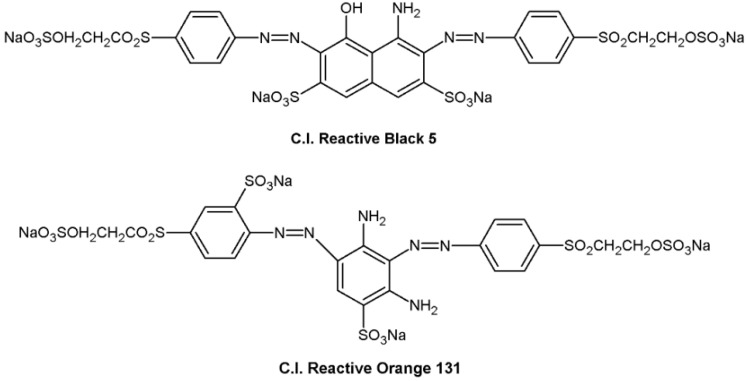
Chemical structure of the anionic dyes used in this study.

Corn starch containing approximately 71% amylopectin and 29% amylose was supplied by Tianjin Tingfung Starch Development Co., Ltd., Tianjin, China. Polyvinyl alcohol (PVA; 1700 degrees of polymerization, 99% hydrolyzed) was obtained from China Petroleum and Chemical Corporation, Shanghai, China. 3-Chloro-2-hydroxypropyltrimethylammonium chloride (CHPTAC) was obtained from Sigma-Aldrich Chemie Gmbh, Steinheim, Germany. Colour Index (C.I.) Reactive Black 5 and C.I. Reactive Orange 131 were supplied by BASF SE, Ludwigshafen, Germany. Their structures are displayed in Figure 1. The commercial activated carbon (AC) was purchased from Ningxia Huahui Activated Carbon Company (Yinchuan, China), and its methylene blue number was 225.3 mg/g. All of the other chemicals were of analytical grade, obtained from Sinopharm Chemical Reagent Co., Ltd. (Shanghai, China), without further purification. 

### 2.2. Preparation of Modified Starch/PVA Composites

Cross-linked cationic PVA (CCP) was obtained by a two-stage process. First, the cationic PVA (CP) was prepared by PVA with CHPTAC, in the presence of sodium hydroxide, at 40 °C for 4 h [40]. In the second step, 7.2 g of Span-80 was added into 100 g of cyclohexane and was stirred, and was then added into 50 g of a cationic PVA aqueous solution (5 wt%). The mixture was homogenized at 6000 r/min by a high-shear dispersion homogenizer for 30 min to form a stable W/O emulsion system. Then, 12 g of a borax solution (2.5 wt%) was dropwise added into the mixture within 30 min, and continued to react for another 4 h at 35 °C. Finally, it was centrifuged, washed with acetone, and dried in an oven at 40 °C. Thus, the CCP microspheres were obtained. The degree of substitution of synthesized CP was 0.24 (2.99 mmol/g of the cationic group content), which was calculated based on the content of the element nitrogen. Cross-linked cationic starch (CCS) and cationic starch (CS) were prepared using borax and CHPTAC as a cross-linking agent and cationic reagent, respectively, in the same way as the above methods. The degree of substitution of the synthesized CS was 0.39 (1.76 mmol/g of the cationic group content), calculated from the nitrogen content. 

The modified starch/PVA composite sample (denoted as CCSP1) was prepared based on the preparation procedures of CCP, by replacing only the CP with a mixture of CS and CP (CS/CP with a mass ratio of 2:1). The same treatments were repeated in order to obtain the samples of CCSP2 and CCSP3, by changing only the mass ratios of CS/CP from 2:1 to 1:1 and 1:2, respectively. 

### 2.3. Characterization 

The FT-IR spectra of the samples were measured using a Nicolet IS 10 FT-IR spectrophotometer (Nicolet, Madison, Wis., USA). The particle size analyses were carried out using a laser light-scattering-based particle sizer (Rise-2028, Jinan, China), and ethyl alcohol and distilled water were used as the dispersion medium. The sample microtopography was observed using a scanning electron microscope (SEM; JSM-6400, Tokyo, Japan), and the morphology was observed using a transmission electron microscopy (TEM; JEOL JEM-2100F, Tokyo, Japan). The samples were treated with supersonic dispersion before the TEM measurement [41]. The crystalline structure of the samples was examined using powder X-ray diffraction (XRD; DX2700, Dandong, China). The surface charges of the samples were obtained by a Zeta potential analyzer (Malvern-Zetasizer nano ZS, Malvern, UK) under neutral conditions.

### 2.4. Adsorption Studies 

The adsorption properties of the samples were investigated using a fixed-bed column (1.2 cm in diameter × 30 cm in height) connected to a spectrophotometer detector. The fixed-bed column was prepared according to the method of Zhang et al. [42]. In view of this, the microsphere samples (CCS, CCP, and the CCSPs) had some of the swelling properties of water absorption, and diatomite or activated carbon were chosen as the filter aid to increase the space velocity of the fixed-bed column. The column was filled with a mixture adsorbent composed of microsphere samples (5 g) and diatomite (5 g; as the filter aid), or a mixture adsorbent composed of microsphere samples (5 g) and activated carbon (5 g), or only 10 g of activated carbon. The microsphere samples, diatomite, and commercial activated carbon were all sieved into a particle size range of 200–300 mesh. The simulated wastewater was continuously fed to the top of the column at a flow rate of 2 mL/min, which was controlled by a peristaltic pump at room temperature. The absorbance readings were taken at set time intervals to analyze the solutions leaving the column. Among these, iodine and potassium iodide solutions and iodine-boric acid solutions were used as color developing agents for solutions containing starch and PVA, respectively. The simulated wastewater used in this study included dye wastewater (0.5 g/L), starch solution (0.1 g/L), PVA solution (0.1 g/L), and simulated textile wastewater (0.5 g/L of Reactive Orange 131, 0.1 g/L of starch, and 0.1 g/L of PVA). The volumes of the completely purified effluent solution were recorded. The adsorption capacities of the adsorbents were calculated based on the volumes of the completely purified effluent. As for the simulated textile wastewater, the adsorption capacity was the sum of the adsorption amount for each component. The effect of the bed height (9.5, 7.6, and 5.7 cm) and flow rate (2, 3, and 4 mL/min) on the column adsorption capacity of the mixture adsorbent (CCSP2 and AC) to treat the textile wastewater was investigated. The effluent solution was analyzed to yield output concentration breakthrough curves.

### 2.5. The Recycle of Disabled Adsorbent 

After adsorption, the disabled mixture adsorbent was recycled as follows. First, the disabled adsorbent was dried at 110 °C for 12 h under an air atmosphere, and then carbonized at 350 °C for 3 h under an N_2_ atmosphere. Next, the carbon spheres were impregnated with the activating agent KOH [43], and the activation process was carried out at 850 °C for 2 h under an N_2_ atmosphere. The obtained material, signed as regenerated activated carbon (RAC), was washed with diluted HCl and deionized water separately, and finally dried. 

**Figure 2:**
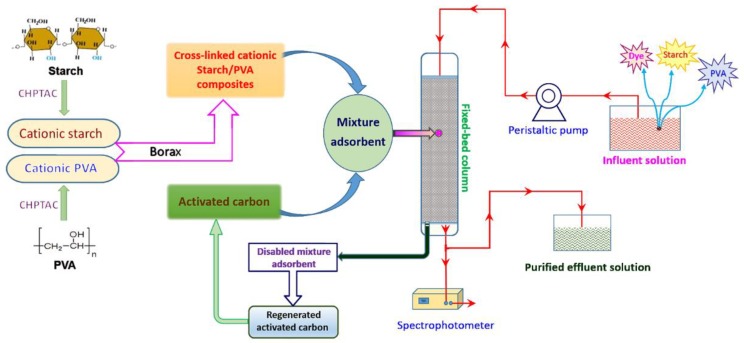
Schematic representation of the adsorption-recycle process. PVA—polyvinyl alcohol; CHPTAC—3-chloro-2-hydroxypropyltrimethylammonium chloride.

The schematic representation of the adsorption-recycle process is shown in Figure 2.

## 3. Results

### 3.1. FT-IR Analysis

**Figure 3:**
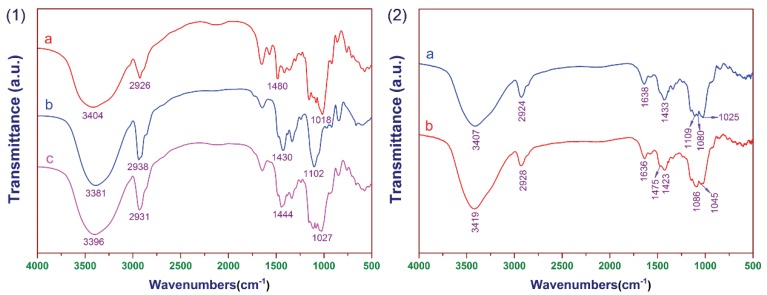
(**1**) The FT-IR spectra of (**a**) cross-linked cationic starch (CCS), (**b**) cross-linked cationic PVA (CCP), and (**c**) CCSP with a mass ratio of CS/CP of 1:1 (CCSP2). (**2**) The FT-IR spectra of the mixture adsorbent was composed of CCSP2 and activated carbon (AC), (**a**) before and (**b**) after treating the textile wastewater.

The FT-IR spectra of samples are shown in Figure 3(1). It can be seen that the main absorption bands for CCSP2, as depicted in curve c, were found at 3396, 2931, 1444, and 1027 cm^−1^. The peak at 3396 cm^−1^ was attributed to the stretching vibration of hydroxyl. The peak at 2931 cm^−1^ was caused by the stretching vibration of C–H. The peak at 1027 cm^−1^ was attributed to the stretching vibration of C–O–C. The peak at 1444 cm^−1^ was assigned to the bending vibration of C–N [32,44]. For the FT-IR spectra of (a) CCS, the peaks at 3404, 2926, and 1018 cm^−1^ were attributed to the stretching vibration of O–H, C–H, and C–O–C, respectively. The peak at 1430 cm^−1^ in curve b (CCP) was similarly assigned to the bending vibration of C–N. The FT-IR spectra of the mixture adsorbent composed of CCSP and AC before and after treating the textile wastewater are shown in Figure 3(2); it can be seen that the characteristic peak of the hydroxyl group (3419 cm^−1^) was enhanced relatively after treating the textile wastewater, and the peak at 1475 cm^−1^ confirmed the existence of azo groups in the dye molecules.

### 3.2. XRD Analysis

**Figure 4:**
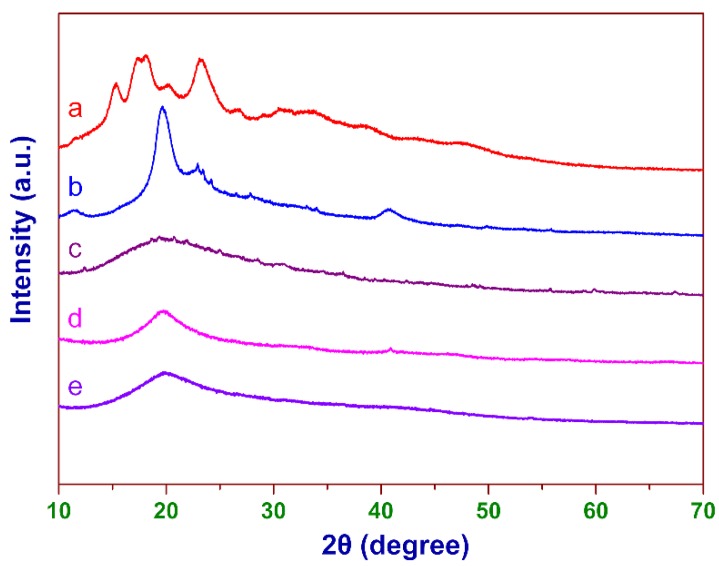
The XRD patterns of (**a**) starch, (**b**) PVA, (**c**) CCS, (**d**) CCP, and (**e**) CCSP2.

As shown in Figure 4, it can be clearly seen that corn starch exhibited a typical A-type XRD pattern [45], and strong diffraction bands centered at about 15°, 17°, 18°, and 23° (2*θ* degrees). However, the XRD pattern of CCS had only a dispersed broad peak, and the crystallization peaks of starch disappeared (see curve c), indicating that the modification could inhibit the crystallization of starch molecules [45]. As for PVA, the intensity of its crystallization peak also significantly decreased after cross-linking and cationic modification. The XRD pattern of CCSP2 was similar to that of CCP.

### 3.3. SEM and TEM Analysis

**Figure 5:**
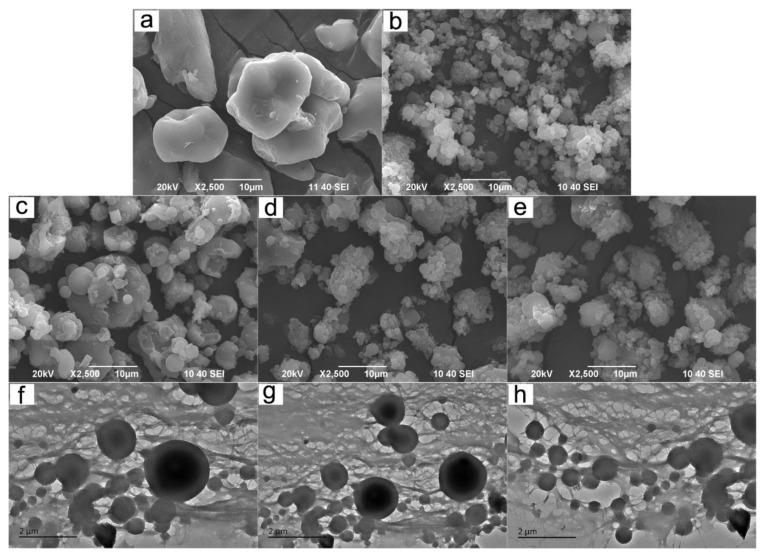
SEM images of (**a**) CCS, (**b**) CCP, (**c**) CCSP1, (**d**) CCSP2, and (**e**) CCSP with a mass ratio of CS/CP of 1:2 (CCSP3). TEM images of (**f**) CCSP1, (**g**) CCSP2, and (**h**) CCSP3.

From the SEM images (Figure 5a–e), the particle sizes of CCS were larger than those of the other four samples. The morphology of most of the CCP was shaped with a good sphericity, although some particles congregated together. It can be obviously seen that the particle sizes of the CCSPs were all larger than that of CCP, but smaller than that of CCS, and gradually increased with the dosage increase of cationic starch in its preparation. This result was in good agreement with the analysis of the TEM images (see Figure 5f–h).

### 3.4. Particle Size Analysis

**Figure 6:**
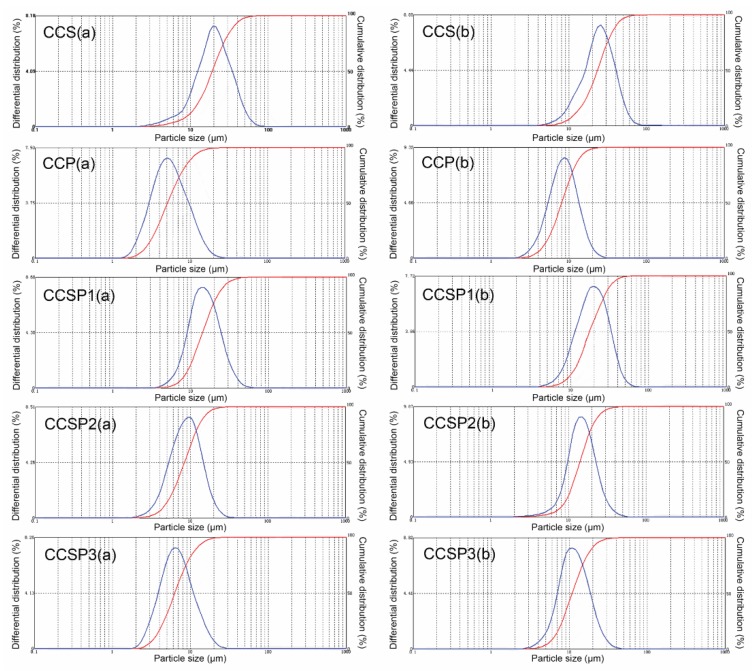
Particle size distribution of samples by using (**a**) ethyl alcohol or (**b**) distilled water as the dispersion medium.

The particle size distributions of CCS, CCP, and the CCSPs are shown in Figure 6. From Figure 6, no matter whether using ethyl alcohol or distilled water as the dispersion medium, the average diameter of CCS was the largest, while the average diameter of CCP was the smallest out of the five samples. This result was in good agreement with the morphology analyses of the samples (see Figure 5). The average diameters of the five kinds of microsphere samples all increased obviously when the dispersion medium was changed from ethyl alcohol to distilled water, indicating that CCS, CCP, and the CCSPs all had some of the swelling properties of water absorption. This result meant that the microsphere samples should be used together with filter aids (diatomite or activated carbon) in the column adsorption process. 

### 3.5. Application in Wastewater Treatment

The adsorption properties of the samples were investigated using the fixed-bed column. Previous research has shown that the cationic group built-in CCS molecule could interact with dye molecules in wastewater via charge neutralization [46]. The results of the fixed-bed column adsorption experiments are shown in Table 1. Among these, the adsorption capacities of CCS, CCP, the CCSPs were calculated by subtracting the adsorption capacity of diatomite from that of the corresponding mixture adsorbents.

From Table 1, CCS, CCP, and the CCSPs all had a much larger adsorption capacity than that of AC when treating simulated dye wastewater, and CCP had the largest dye adsorption capacity, owing to its highest cationic group content. Meanwhile, they all had a smaller adsorption capacity than that of AC when treating a starch or PVA solution. CCS had a better adsorption capacity than that of CCP and the CCSPs when treating a starch solution, but CCP had a better adsorption capacity than that of CCS and CCSPs when treating a PVA solution. All of the CCSPs had a larger adsorption capacity than that of CCS, and had a smaller adsorption capacity than that of CCP when treating the simulated dye wastewater and PVA solution. Inversely, all of the CCSPs had a smaller adsorption capacity than that of CCS, and had a larger adsorption capacity than that of CCP when treating a starch solution. The composition of the CCSPs had a great influence on their adsorption capacity. The adsorption capacities of the CCSPs for Reactive Black 5, Reactive Orange 131, and PVA all gradually increased with the dosage increase of cationic PVA in their preparation, but decreased for starch. These results indicated that the adsorption capacities of the CCSPs could be developed based on the respective advantages of CCS and CCP when they were used to treat practical textile wastewater. Compared with CCS and CCP, the adsorption capacity of the CCSPs increased significantly when treating simulated textile wastewater. The adsorption capacities of the CCSPs were over 10 times larger than that of the commercial activated carbon (AC) when treating dye wastewater. According the zeta potential analysis (see Table 2), the surfaces of the CCSPs all possessed a high positive charge, whereas the dye molecules had negative charges in the solutions. This means an electrostatic interaction between the cationic group built-in CCSP and dye molecules there should exist [46]. In addition, CCSP had quite a similar molecular structure, with both the starch and PVA molecules, indicating that a hydrogen bonding mechanism might also exist. Therefore, the electrostatic interaction and hydrogen-binding interaction might be the main adsorption mechanisms of CCSP for treating textile wastewater.

**Figure 7:**
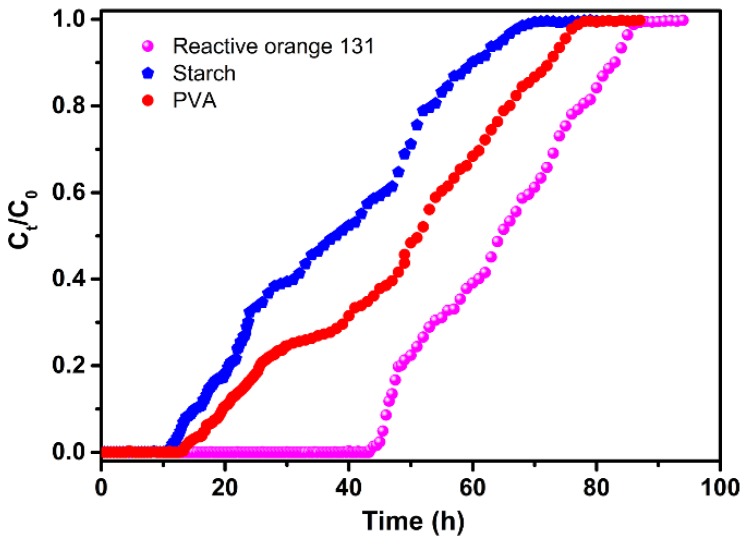
Breakthrough curves in the fixed-bed column. Experimental operating conditions: the mixture adsorbent composed of CCSP2 and AC (mass ratio of 1:1; 10 g), flow rate = 2 mL/min.

It is worthwhile to note that the mixture adsorbent composed of CCSP2 and AC (1:1 in weight) had the largest adsorption capacity compared with the other adsorbents when treating the simulated textile wastewater, which indicated that the combination of AC and CCSP2 could show an obvious synergetic effect on each other for adsorption, and that the starch, PVA, and dyes could be removed effectively from the simulated textile wastewater simultaneously. The effect of the bed height and flow rate on the adsorption capacity of the mixture adsorbent (CCSP2 and AC) to treat the textile wastewater was investigated, and the results are shown in Table 3. It can be found that the column adsorption performance of the mixture adsorbent could be enhanced at a higher bed height and lower flow rate. The reason for this could be that the residence time of the adsorbate molecules in fixed-bed columns could be longer at the higher bed height and at the lower flow rate, and more adsorbate molecules would establish the adsorption equilibrium in the column [18,25]. As shown in Figure 7, the breakthrough curves of the dye, starch, and PVA were diverse from each other, and the breakthrough point, defined as the point at which the pollutant concentration (any one of starch, or PVA, or dyes) of the effluent reached to above zero, of the starch first appeared, while at this point, the mixture adsorbent still had a high adsorption capacity to the dye and PVA. This result indicated that it could obtain the higher adsorption ability for treating textile wastewater by adjusting the composition of the mixture adsorbent based on the breakthrough curves of the adsorbates.

### 3.6. The Recycle of Disabled Adsorbent

**Figure 8:**
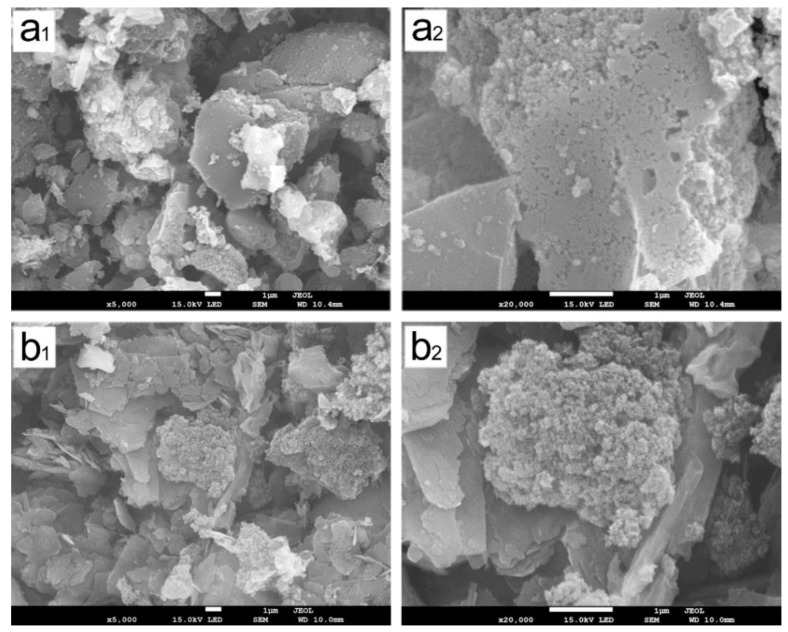
SEM images of the AC (**a_1_** and **a_2_**) and regenerated activated carbon (**b_1_** and **b_2_**).

**Figure 9:**
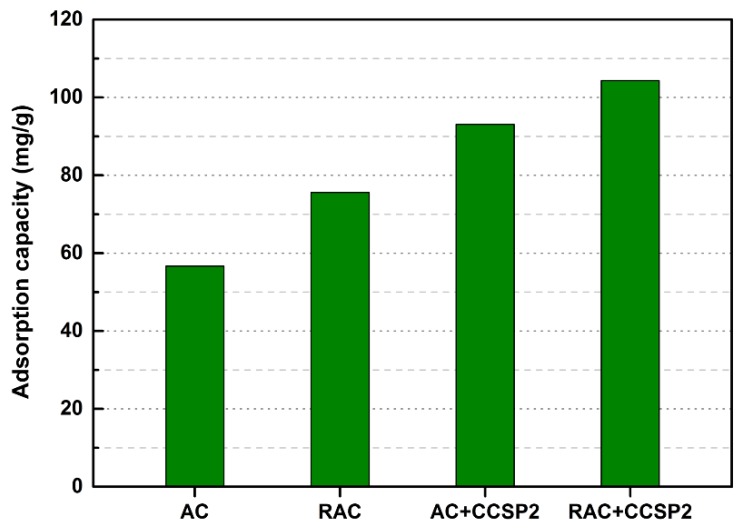
The adsorption capacities of the AC and RAC to the simulated textile wastewater in column.

After treating the simulated textile wastewater, the particle surfaces of the mixture adsorbent composed of CCSP and AC would gather a large amount of starch, PVA, and dyes. It was difficult to be regenerated by the conventional desorption methods. In order to avoid producing solid waste, a chemical activation process was carried out to convert these disabled adsorbents into regenerated activated carbon. The SEM images of the original activated carbon (AC) and the regenerated activated carbon (RAC) that came from the disabled mixture adsorbent composed of CCSP2 and AC (mass ratio of 1:1) are shown in Figure 8. As can be seen from Figure 8, significant differences in the morphology of the RAC and AC were observed. The average particle size of the RAC was obviously smaller than that of the AC, and some honeycomb-shaped porous carbons were developed after carbonization. The Brunauer-Emmett-Teller (BET) data (see Table 4) showed that the RAC had a larger surface area and pore volume than that of AC. This result indicated that the starch and PVA in CCSP2 should be converted into biomass carbon and porous carbon after carbonization [47,48]. It is worth mentioning that the adsorption capacity of RAC on the simulated textile wastewater was higher than that of the original activated carbon, no matter whether used alone or in combination with CCSP2 (see Figure 9). This result indicated that the mixture adsorbent composed of CCSP2 and AC exhibited the advantages of a high adsorption capacity, recycling ability, and no solid waste when it was used as the adsorbent of the fixed-bed to treat the simulated textile wastewater.

## 4. Conclusions

A novel modified starch/polyvinyl alcohol composite (CCSP) was prepared and employed as a highly efficient adsorbent for textile wastewater treatment. The CCSP was shaped with sphericity, and had some of the swelling properties of water absorption and a lower crystallinity. The dye adsorption capacity of CCSP was much larger than that of AC. The mixture adsorbent composed of CCSP and AC could make full use of the respective advantages of its components, and could remove starch, PVA, and dyes from the textile wastewater simultaneously, combined with the fixed-bed adsorption technique. The fixed-bed column adsorption process could be influenced by the bed height and flow rate. After adsorption, the disabled mixture adsorbent was converted into regenerated activated carbon for sustainable use by the chemical activation process, and the regenerated activated carbon had a higher adsorption capacity to the textile wastewater than AC. The developed method, through fixed-bed adsorption using CCSP, would be an excellent green textile wastewater treatment, on account of the high adsorption capacity, recycling ability, and no solid waste.

## Figures and Tables

**Table 1:** The results of the fixed-bed column adsorption experiments.

Simulated Wastewater Solutions	The Adsorption Capacity (mg/g)						
	CCS	CCP	CCSP1	CCSP2	CCSP3	AC	Mixture Adsorbent (CCSP2 and AC)
Reactive Black 5	572.0	693.5	605.0	629.5	661.0	54.0	378.0
Reactive Orange 131	494.0	605.5	539.0	560.0	573.5	48.0	340.5
Starch solution	12.4	9.3	11.4	10.6	9.9	16.2	14.4
PVA solution	7.7	16.2	10.1	13.4	15.1	19.6	17.7
Textile wastewater	40.6	52.5	68.6	73.5	60.9	56.7	93.1

**Table 2:** The zeta potential of the prepared microspheres.

Adsorbents	Zeta Potential (mV)
CCS	20.3
CCP	30.4
CCSP1	22.7
CCSP2	26.2
CCSP3	28.1

**Table 3:** Effect of bed height and flow rate on the column adsorption capacity of the mixture adsorbent (CCSP2 and AC) to treat the textile wastewater.

Bed Height (cm)	Flow Rate (mL/min)	The Adsorption Capacity (mg/g)
9.5 (10g)	2	93.1
7.6 (8g)	2	88.9
5.7 (6g)	2	80.5
9.5 (10g)	3	86.8
9.5 (10g)	4	77.0

**Table 4:** Characterization of the regenerated activated carbon (RAC) and AC.

Characterization	BET Surface Area (m^2^/g)	Pore Volume (cm^3^/g)	Average Pore Diameter (nm)
AC	386	0.39	4.12
RAC	581	0.53	3.13

## References

[1] Mirzaie M., Rashidi A., Tayebi H.A., Yazdanshenas M.E. (2018). Optimized removal of acid blue 62 from textile waste water by SBA-15/PAMAM dendrimer hybrid using response surface methodology. J. Polym. Environ..

[2] Pekakis P.A., Xekoukoulotakis N.P., Mantzavinos D. (2006). Treatment of textile dyehouse wastewater by TiO_2_ photocatalysis. Water Res..

[3] Rauf M.A., Ashraf S.S. (2009). Fundamental principles and application of heterogeneous photocatalytic degradation of dyes in solution. Chem. Eng. J..

[4] Liu R.R., Lu X.J., Tian Q., Yang B., Chen J.H. (2011). The performance evaluation of hybrid anaerobic baffled reactor for treatment of PVA-containing desizing wastewater. Desalination.

[5] Sun W.H., Chen J., Chen L.J., Wang J.L., Zhang Y.M. (2016). Coupled electron beam radiation and MBR treatment of textile wastewater containing polyvinyl alcohol. Chemosphere.

[6] Dotto J., Fagundes-Klen M.R., Veit M.T., Palácio S.M., Bergamasco R. (2019). Performance of different coagulants in the coagulation/flocculation process of textile wastewater. J. Clean. Prod..

[7] Jegatheesan V., Pramanik B.K., Chen J., Navaratna D., Chang C.Y., Shu L. (2016). Treatment of textile wastewater with membrane bioreactor: A critical review. Bioresour. Technol..

[8] Cardoso J.C., Bessegato G.G., Zanoni M.V.B. (2016). Efficiency comparison of ozonation, photolysis, photocatalysis and photoelectrocatalysis methods in real textile wastewater decolorization. Water Res..

[9] He P.Y., Zhang Y.J., Chen H., Liu L.C. (2019). Development of an eco-efficient CaMoO_4_/electroconductive geopolymer composite for recycling silicomanganese slag and degradation of dye wastewater. J. Clean. Prod..

[10] Teh C.Y., Budiman P.M., Shak K.P.Y., Wu T.Y. (2016). Recent advancement of coagulation-flocculation and its application in wastewater treatment. Ind. Eng. Chem. Res..

[11] Hsu L.J., Lee L.T., Lin C.C. (2011). Adsorption and photocatalytic degradation of polyvinyl alcohol in aqueous solutions using P-25 TiO_2_. Chem. Eng. J..

[12] Ye B., Li Y., Chen Z., Wu Q.Y., Wang W.L., Wang T., Hu H.Y. (2017). Degradation of polyvinyl alcohol (PVA) by UV/chlorine oxidation: Radical roles, influencing factors, and degradation pathway. Water Res..

[13] Marušincová H., Husárová L., Růžička J., Ingr M., Navrátil V., Buňková L., Koutny M. (2013). Polyvinyl alcohol biodegradation under denitrifying conditions. Int. Biodeterior. Biodegrad..

[14] Paz A., Carballo J., Pérez M.J., Domínguez J.M. (2017). Biological treatment of model dyes and textile wastewaters. Chemosphere.

[15] Zhang Y., Xia K., Liu X., Chen Z., Du H., Zhang X. (2019). Synthesis of cationic-modified silica gel and its adsorption properties for anionic dyes. J. Taiwan Inst. Chem. Eng..

[16] Xia L., Li C., Zhou S., Fu Z., Wang Y., Lyu P., Zhang J., Liu X., Zhang C., Xu W. (2019). Utilization of waste leather powders for highly effective removal of dyes from water. Polymers.

[17] Afshari M., Dinari M. (2020). Synthesis of new imine-linked covalent organic framework as high efficient absorbent and monitoring the removal of direct fast scarlet 4BS textile dye based on mobile phone colorimetric platform. J. Hazard. Mater..

[18] Saeed A., Sharif M., Iqbal M. (2010). Application potential of grapefruit peel as dye sorbent: Kinetics, equilibrium and mechanism of crystal violet adsorption. J. Hazard. Mater..

[19] Jain M., Garg V.K., Kadirvelu K., Sillanpää M. (2015). Combined effect of sunflower stem carbon-calcium alginate beads for the removal and recovery of chromium from contaminated water in column mode. Ind. Eng. Chem. Res..

[20] Zhao L., Basly J.P., Baudu M. (2017). Macroporous alginate/ferrihydrite hybrid beads used to remove anionic dye in batch and fixed-bed reactors. J. Taiwan Inst. Chem. Eng..

[21] Jain M., Garg V.K., Kadirvelu K. (2013). Cadmium (II) sorption and desorption in a fixed bed column using sunflower waste carbon calcium-alginate beads. Bioresour. Technol..

[22] Kurade M.B., Waghmode T.R., Xiong J.Q., Govindwar S.P., Jeon B.H. (2019). Decolorization of textile industry effluent using immobilized consortium cells in upflow fixed bed reactor. J. Clean. Prod..

[23] Demarchi C.A., Campos M., Rodrigues C.A. (2013). Adsorption of textile dye Reactive Red 120 by the chitosan-Fe (III)-crosslinked: Batch and fixed-bed studies. J. Environ. Chem. Eng..

[24] Faki A., Turan M., Ozdemir O., Turan A.Z. (2008). Analysis of fixed-bed column adsorption of reactive yellow 176 onto surfactant-modified zeolite. Ind. Eng. Chem. Res..

[25] Gong J.L., Zhang Y.L., Jiang Y., Zeng G.M., Cui Z.H., Liu K., Deng C.H., Niu Q.Y., Deng J.H., Huan S.Y. (2015). Continuous adsorption of Pb (II) and methylene blue by engineered graphite oxide coated sand in fixed-bed column. Appl. Surf. Sci..

[26] Gomez V., Larrechi M.S., Callao M.P. (2007). Kinetic and adsorption study of acid dye removal using activated carbon. Chemosphere.

[27] Mahmoodi N.M., Salehi R., Arami M. (2011). Binary system dye removal from colored textile wastewater using activated carbon: Kinetic and isotherm studies. Desalination.

[28] Silva T.L., Ronix A., Pezoti O., Souza L.S., Leandro P.K.T., Bedin K.C., Beltrame K.K., Cazetta A.L., Almeida V.C. (2016). Mesoporous activated carbon from industrial laundry sewage sludge: Adsorption studies of reactive dye Remazol Brilliant Blue R. Chem. Eng. J..

[29] Jain M., Yadav M., Kohout T., Lahtinen M., Garg V.K., Sillanpää M. (2018). Development of iron oxide/activated carbon nanoparticle composite for the removal of Cr (VI), Cu (II) and Cd (II) ions from aqueous solution. Water Resour. Ind..

[30] Miculescu F., Maidaniuc A., Voicu S.I., Thakur V.K., Stan G.E., Ciocan L.T. (2017). Progress in hydroxyapatite-starch based sustainable biomaterials for biomedical bone substitution applications. ACS Sustain. Chem. Eng..

[31] Domene-López D., García-Quesada J.C., Martin-Gullon I., Montalbán M.G. (2019). Influence of starch composition and molecular weight on physicochemical properties of biodegradable films. Polymers.

[32] Pal S., Mal D., Singh R.P. (2005). Cationic starch: An effective flocculating agent. Carbohydr. Polym..

[33] Prado H.J., Matulewicz M.C. (2014). Cationization of polysaccharides: A path to greener derivatives with many industrial applications. Eur. Polym. J..

[34] Simanaviciute D., Klimaviciute R., Rutkaite R. (2017). Equilibrium adsorption of caffeic, chlorogenic and rosmarinic acids on cationic cross-linked starch with quaternary ammonium groups. Int. J. Biol. Macromol..

[35] Koriche Y., Darder M., Aranda P., Semsari S., Ruiz-Hitzky E. (2014). Bionanocomposites based on layered silicates and cationic starch as eco-friendly adsorbents for hexavalent chromium removal. Dalton Trans..

[36] Wu H., Liu Z., Li A., Yang H. (2017). Evaluation of starch-based flocculants for the flocculation of dissolved organic matter from textile dyeing secondary wastewater. Chemosphere.

[37] Dou Y., Zhang B., He M., Yin G., Cui Y., Savina I. (2015). Keratin/Polyvinyl alcohol blend films cross-linked by dialdehyde starch and their potential application for drug release. Polymers.

[38] Liu X., Fatehi P., Ni Y., Xiao H. (2010). Using cationic polyvinyl alcohol (C-PVA) to improve the strength of wood-free papers containing high-yield pulp (HYP). Holzforschung.

[39] Santos A., de Oliveira F.W.F., Silva F.H.A., Maria D.A., Ardisson J.D., de Almeida Macêdo W.A., Palmieri H.E.L., Franco M.B. (2012). Synthesis and characterization of iron-PVA hydrogel microspheres and their use in the arsenic (V) removal from aqueous solution. Chem. Eng. J..

[40] Fatehi P., Xiao H. (2010). Effect of cationic PVA characteristics on fiber and paper properties at saturation level of polymer adsorption. Carbohydr. Polym..

[41] Duan Y., Liu Y., Chen Z., Liu D., Yu E., Zhang X., Fu H., Fu J., Zhang J., Du H. (2020). Amorphous molybdenum sulfide nanocatalysts simultaneously realizing efficient upgrading of residue and synergistic synthesis of 2D MoS_2_ nanosheets/carbon hierarchical structures. Green Chem..

[42] Zhang Y., Li J., Li W. (2015). Effect of particle size on removal of sunset yellow from aqueous solution by chitosan modified diatomite in a fixed bed column. RSC Adv..

[43] Sudaryanto Y., Hartono S.B., Irawaty W., Hindarso H., Ismadji S. (2006). High surface area activated carbon prepared from cassava peel by chemical activation. Bioresour. Technol..

[44] Fang L., Zhang X., Ma J., Sun D., Zhang B., Luan J. (2015). Eco-Friendly cationic modification of cotton fabrics for improving utilization of reactive dyes. RSC Adv..

[45] Wang Y., Xie W. (2010). Synthesis of cationic starch with a high degree of substitution in an ionic liquid. Carbohydr. Polym..

[46] Klimaviciute R., Riauka A., Zemaitaitis A. (2007). The binding of anionic dyes by cross-linked cationic starches. J. Polym. Res..

[47] Shen F., Liu J., Dong Y., Wu D. (2018). Mercury removal by biomass-derived porous carbon: Experimental and theoretical insights into the effect of H_2_S. Chem. Eng. J..

[48] Zhang S.J., Yu H.Q., Feng H.M. (2006). PVA-based activated carbon fibers with lotus root-like axially porous structure. Carbon.

